# Crystal structure of a new hydrate form of the NSAID sodium diclofenac

**DOI:** 10.1107/S2056989020015108

**Published:** 2020-11-20

**Authors:** Ismael Angel Nieto, Sylvain Bernès, Aarón Pérez-Benítez

**Affiliations:** a Instituto de Física, Benemérita Universidad Autónoma de Puebla, 72570 Puebla, Pue., Mexico; b Facultad de Ciencias Químicas, Benemérita Universidad Autónoma de Puebla, 72570 Puebla, Pue., Mexico

**Keywords:** crystal structure, diclofenac, NSAID, hydrate, Hirshfeld analysis

## Abstract

The crystal structure is reported of sodium 2-[2-(2,6-di­chloro­anilino)phen­yl]acetate 3.5-hydrate or tetra-μ-aqua-κ^8^ *O*:*O*-deca­aqua­bis­{μ_3_-2-[2-(2,6-di­chloro­anilino)phen­yl]acetato-κ^3^ *O*:*O*:*O*}tetra­sodium(I) bis­{2-[2-(2,6-di­chloro­anil­ino)phen­yl]acetate}, {[Na_4_(C_14_H_10_Cl_2_NO_2_)_2_(H_2_O)_14_](C_14_H_10_Cl_2_NO_2_)_2_}_*n*_, which re­presents a new hydrate form of the NSAID sodium diclofenac (SD). The triclinic unit cell contains one ionic compound with formula Na_4_(C_14_H_10_Cl_2_NO_2_)_4_(H_2_O)_14_, in which two symmetry-related carboxyl­ate anions C_14_H_10_Cl_2_NO_2_^−^ are bonded to a centrosymmetric [Na_4_]^4+^ core cationic cluster, while the others are only hydrogen bonded to the cationic cluster. The conformation for the anions is similar to that found in other diclofenac compounds, and the [Na_4_(O_carbox_)_2_(H_2_O)_14_]^4+^ cluster displays an unprecedented geometry, which can be described as an incomplete dicubane cluster formed by face-sharing incomplete cubes. A complex framework of O—H⋯O hydrogen bonds stabilizes the crystal structure. The herein reported crystal structure for SD·3.5H_2_O in space group *P*  is different from those previously reported for other hydrates, namely SD·4.75H_2_O (*P*2_1_) and SD·5H_2_O (*P*2_1_/*m*).

## Abstract 2

The crystallization of a new hydrated form of sodium diclofenac was achieved, with the anions surrounding an [Na_4_]^4+^ cationic cluster formed with water mol­ecules and carb­oxy­lic O atoms.

## Chemical context   

Diclofenac (IUPAC name: 2-[2-(2,6-di­chloro­anilino)phen­yl]acetic acid, C_14_H_11_Cl_2_NO_2_), sold under the brand names Voltaren and Ecofenac, among others, is a non-steroidal anti-inflammatory drug (NSAID), with anti­pyretic and analgesic properties. It is prescribed for pain management in chronic inflammatory disorders, like arthritis, rheumatoid arthritis, and osteoarthritis (Sallmann, 1986 ▸). It is available as sodium diclofenac (SD hereafter) or potassium diclofenac, and generated global retail sales of over USD 440 million in 2018.

Diclofenac acid is a polymorphous compound, for which crystal structures have been reported in space groups *C*2/*c* (Moser *et al.*, 1990 ▸; Kovala-Demertzi *et al.*, 1993 ▸; Muangsin *et al.*, 2004 ▸; Niranjana Devi *et al.*, 2019 ▸), *P*2_1_/*c* (Castellari & Ottani, 1997 ▸; Perlovich *et al.*, 2007 ▸; King *et al.*, 2011 ▸) and *Pcan* (Jaiboon *et al.*, 2001 ▸). This acid can also be co-crystallized with small aromatic compounds (*e.g*. Báthori *et al.*, 2011 ▸; Zheng *et al.*, 2019 ▸). Regarding the carboxyl­ate anion, C_14_H_10_Cl_2_NO_2_^−^, it has been extensively used as a ligand for coordination chemistry with transition metals (*e.g*. Sayen & Guillon, 2012 ▸; Bera *et al.*, 2020 ▸). Finally, crystal structures for hydrated alkali salts of diclofenac were established, with Na^+^ (Muangsin *et al.*, 2002 ▸; Llinàs *et al.*, 2007 ▸), K^+^ (Chu & Cheng, 2007 ▸), Ca^2+^ (Duan & Li, 2018 ▸) and Mg^2+^ (Castellari *et al.*, 1999 ▸).

The exact water content of the SD salt used by manufacturers as medicine-grade API remains unclear. Vendors generally refer to the CAS-referenced compound CAS-15307-79-6, and describe the raw material as ‘slightly hygroscopic’. The aforementioned X-ray structures correspond to the penta­hydrate salt, SD·5H_2_O (Muangsin *et al.*, 2002 ▸) and to a slightly less hydrated phase, SD·4.75H_2_O (Llinàs *et al.*, 2007 ▸). In the former study, crystals were obtained by slow evaporation of a mixture of chitosan and SD dissolved in ethyl acetate and aqueous acetic acid. The space group is reported as *P*2_1_/*m*, with two independent diclofenac anions and partially disordered Na^+^ cations and water mol­ecules. In the latter study, single crystals were obtained by recrystallization from ethanol of commercially available anhydrous SD, affording crystals with cell parameters very close to those of the previous study. However, the structure was refined in space group *P*2_1_, with four independent diclofenac anions, and 4.75 water mol­ecules per diclofenac. All sites are fully occupied, and the Na positions are different in both structures.
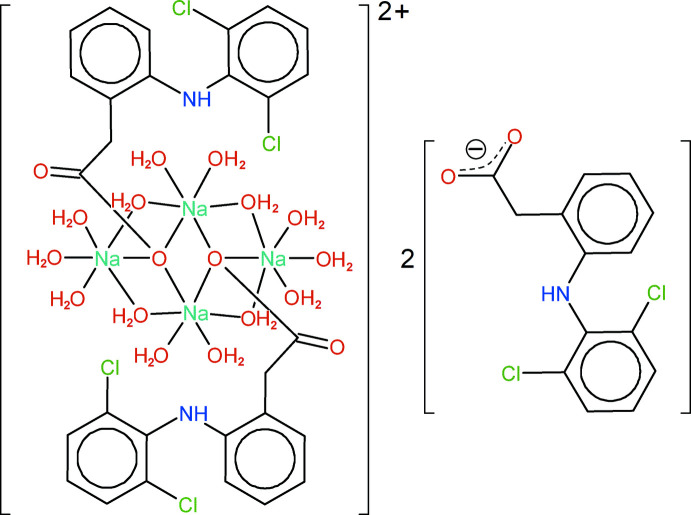


With these results, Llinàs *et al.* (2007 ▸) concluded that in the solid state, the formula of the stable hydrated form of sodium diclofenac should be close to SD·5H_2_O. We now report that a less hydrated form with formula SD·3.5H_2_O can be crystallized in space group *P* 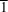, when SD is recrystallized from acetone.

## Structural commentary   

**Figure 1:**
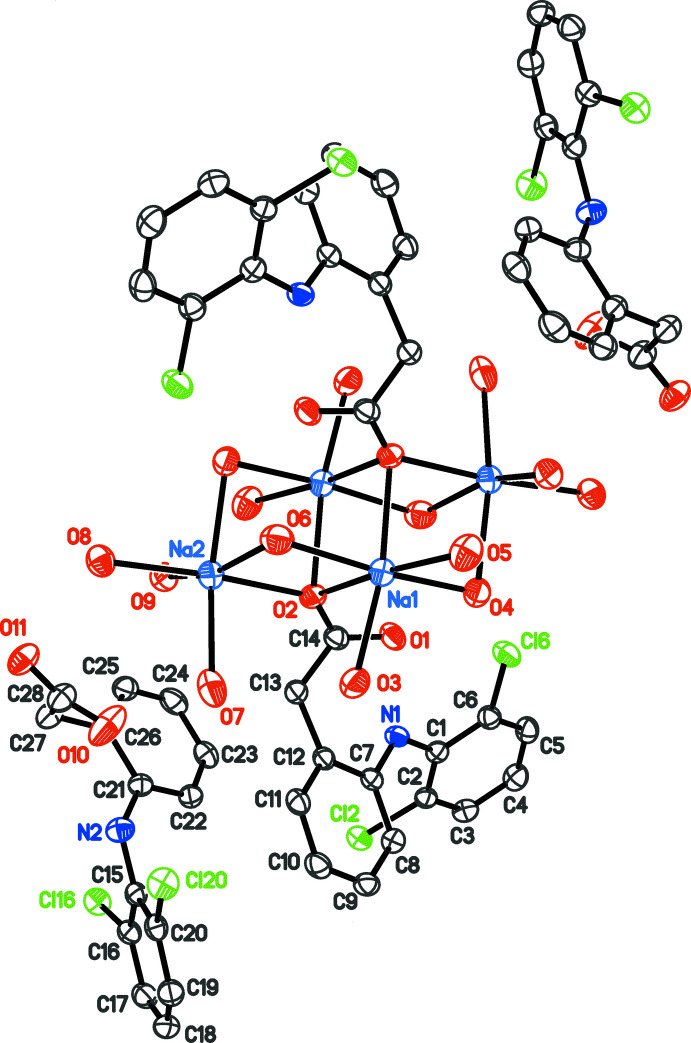
Structure of Na_4_(C_14_H_10_Cl_2_NO_2_)_4_(H_2_O)_14_ (one triclinic unit cell) with displacement ellipsoids for non-H atoms at the 30% probability level. Non-labelled atoms are generated by symmetry code 2 − *x*, −*y*, 1 − *z*.

The asymmetric unit of the triclinic cell contains two diclofenac anions, balanced with two Na^+^ cations. One diclofenac is hydrogen bonded to water mol­ecules, while the other is bridging the Na^+^ cations, with bond lengths Na1—O2 = 2.535 (3) and Na2—O2 = 2.401 (3) Å. The bridge is close to an inversion centre, and then a third Na—O bond is formed, with Na1^i^—O2 = 2.542 (3) Å [symmetry code: (i) 2 − *x*, −*y*, 1 − *z*]. The resulting μ_3_ bridging mode of the diclofenac anion is very uncommon. The triply bridging O-atom mode is well known in metal alkoxides, but very rare for carboxyl­ates (Wu & Mak, 1996 ▸), and found almost exclusively in polymeric compounds. This bridging mode was not observed in the previously reported SD hydrates. The asymmetric unit is completed with seven water mol­ecules bonded to the Na^+^ cations at distances ranging from 2.387 (3) to 2.608 (4) Å. One water mol­ecule, O6, bridges the Na^+^ cations, while all others are in terminal positions on their carrier sites. Once the crystallographic inversion centre operates to form the complete structure, the unit-cell content is Na_4_(C_14_H_10_Cl_2_NO_2_)_4_(H_2_O)_14_ (Fig. 1 ▸). The compound formula may be reduced to the minimal chemical formula SD·3.5H_2_O. There is no evidence of disorder in the mol­ecular structure.

Although numerous Na/O/H_2_O clusters are reported in the literature, the Na-based framework that holds together the four diclofenac ions in the unit cell is only found as a sub-framework in a few structures with higher complexity, such as dicubanes (Song *et al.*, 2007 ▸). The [Na_4_(O_carbox_)_2_(H_2_O)_14_]^4+^ cluster, which includes two carboxyl­ate O atoms from the coordinated diclofenac anions, can be described as an incomplete dicubane cluster formed by face-sharing incomplete cubes. All Na centres are six-coordinate, with distorted octa­hedral geometry and *cis* O—Na—O angles in the range 79.41 (10) to 115.21 (10)°.

The two independent diclofenac ions display similar conformations, characterized by the dihedral angle formed by the benzene rings, 54.2 (1) and 58.9 (1)°. This conformation falls within the expected range of dihedral angles: for 151 structures retrieved from the CSD including the diclofenac anion, the benzene–benzene dihedral angles span the range 54.3 to 89.0° (Groom *et al.*, 2016 ▸). This bent conformation results from the rotational barrier imposed by the Cl atoms, and is not influenced by the presence of the core [Na_4_(O_carbox_)_2_(H_2_O)_14_]^4+^ cluster. This conformation is also stabilized *via* intra­molecular N—H⋯O hydrogen bonds of moderate strength, between the amine and carboxyl­ate groups (Table 1 ▸, entries 1 and 2). The torsion between the aromatic rings is indeed recognized as a factor related to the biological properties of diclofenac (Menassé *et al.*, 1978 ▸; Sallmann, 1986 ▸).

## Supra­molecular features   

**Figure 2:**
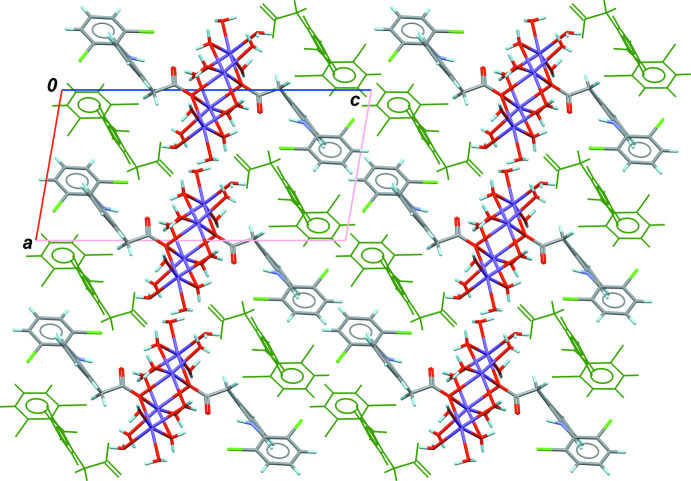
Part of the crystal structure of SD·3.5H_2_O viewed along the crystallographic *b* axis. Green anions are those which are not coordinated to the [Na_4_(O_carbox_)_2_(H_2_O)_14_]^4+^ cluster.

**Figure 3:**
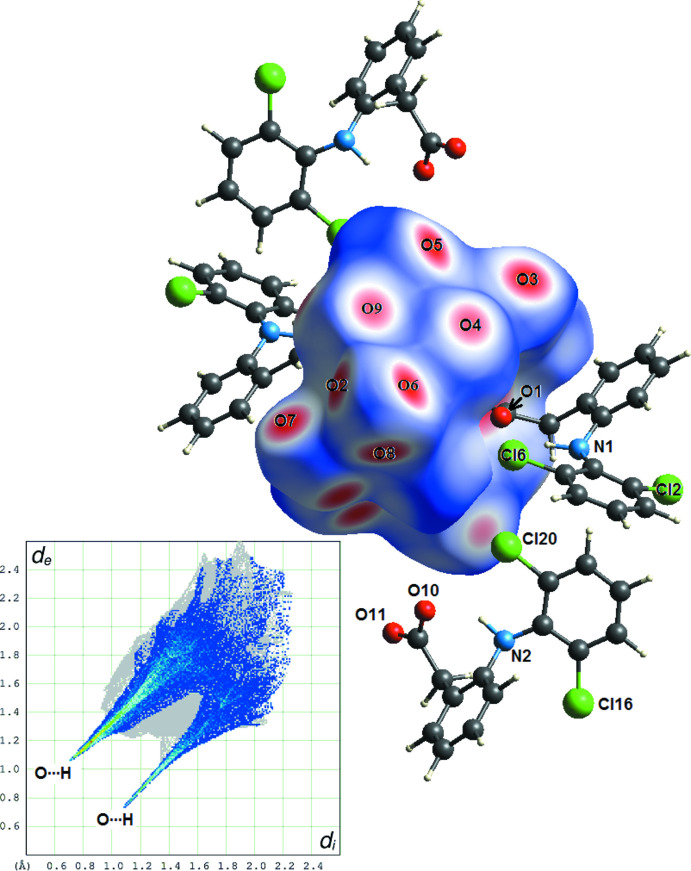
Hirshfeld surface (Turner *et al.*, 2017 ▸) calculated for the [Na_4_(H_2_O)_14_]^4+^ framework. The surface is mapped over *d*_norm_ (−0.66 to 1.41 Å) and the four diclofenac anions completing the unit cell are also represented. Each labelled bright-red patch on the surface is associated with an O atom (water or carboxyl­ate group) involved in hydrogen bonds. In the *d*_e_ *vs d*_i_ fingerprint plot, coloured pixels are for O⋯H and H⋯O contacts.

The crystal packing is quite efficient, with a high Kitaigorodskii packing index of 0.72, even in the absence of π–π inter­actions (Spek, 2020 ▸). The [Na_4_(H_2_O)_14_(C_14_H_10_Cl_2_NO_2_)_2_]^2+^ cations are well separated in the crystal by uncoord­inated anions (C_14_H_10_Cl_2_NO_2_)^−^, leaving no room for free water mol­ecules (Fig. 2 ▸). All water mol­ecules are linked to the central Na_4_ cluster and participate broadly in the stabilization of the crystal structure, *via* classical O—H⋯O hydrogen bonds (Table 1 ▸), clearly visible on the Hirshfeld map build-up on the [Na_4_(H_2_O)_14_]^4+^ framework (Fig. 3 ▸; Turner *et al.*, 2017 ▸). The contribution of O⋯H/H⋯O contacts is predominant (39.3%) for crystal cohesion, and the sharp spikes in the fingerprint plot at *d*_i_ + *d*_e_ ≃ 2.1 Å are typical of effective water⋯water and water⋯carboxyl­ate inter­actions. A secondary inter­action is observed, accounting for 8.8% of the Hirshfeld map, which corresponds to inter­molecular O—H⋯Cl bonds involving one terminal water mol­ecule in the [Na_4_(O_carbox_)_2_(H_2_O)_14_]^4+^ cluster (Table 1 ▸, last entry). As a consequence of the high density of water mol­ecules in the [Na_4_(O_carbox_)_2_(H_2_O)_14_]^4+^ cluster, two water H atoms do not form any hydrogen bonds (H62 and H92). However, it is difficult to assess if all water mol­ecules are correctly oriented in our model, since the refinement is based on room-temperature data limited to *d*_min_ = 0.80 Å.

The complete 3D hydrogen-bonding scheme for the whole structure is complex, and obviously very different from supra­molecular structures observed in the previously reported SD hydrates (Muangsin *et al.*, 2002 ▸; Llinàs *et al.*, 2007 ▸). These differences, resulting from the arrangement of water mol­ecules in the crystal, could be relevant regarding the actual bioavailability of SD *in vivo* (Llinàs *et al.*, 2007 ▸). On the other hand, the actual formula of the API used by manufacturers remains unclear, at least with respect to the hydration status. Even some variability of the API formula from one brand to another cannot be excluded. Moreover, we believe that other stable hydrates could be crystallized from commercial SD. Indeed, we did not evaluate the influence of the excipients extracted with SD nor purity of the acetone used for extraction, on the crystallization of the new hydrate.

## Synthesis and crystallization   

Commercial Volfenac Retard was used (*Productos farma­céuticos Collins*, Mexico). Each tablet weighs *ca* 433 mg and includes 100 mg of the API. Main excipients are sucrose, and a small amount of magnesium stearate. One tablet was crushed in a mortar, the resulting fine powder was dispersed in acetone (40 mL) at room temperature, and then filtered over a Büchner funnel. The yellow solution was left at room temperature for slow evaporation of solvent, affording yellow prismatic single crystals suitable for X-ray diffraction.

## Refinement details   

Crystal data, data collection and structure refinement details are summarized in Table 2 ▸. Apparently, all studied single crystals were twinned by rotation around reciprocal axis *b* ^*^. As a consequence, the unit-cell parameters emulate a monoclinic symmetry, with *α* ≃ *γ* ≃ 90° (Table 2 ▸). However, diffraction intensities are not consistent with the 2/*m* Laue group. The structure was then refined with the twin matrix [−1 0 0, 0 1 0, 0 0 −1], and the batch scale factor converged to 0.168 (1). Almost all H atoms bonded to N or O atoms were found in difference maps. Amine H atoms (H1, H2) were refined with free coordinates. Water H atoms were allowed to ride on their O sites, while the water mol­ecules were allowed to rotate about the Na—O bonds (command AFIX 7, Sheldrick, 2015*b* ▸). Other H atoms were refined using a riding model.

## Supplementary Material

Crystal structure: contains datablock(s) I, global. DOI: 10.1107/S2056989020015108/jy2003sup1.cif

Structure factors: contains datablock(s) I. DOI: 10.1107/S2056989020015108/jy2003Isup2.hkl

Supporting information file. DOI: 10.1107/S2056989020015108/jy2003Isup3.smi

CCDC reference: 2044232

Additional supporting information:  crystallographic information; 3D view; checkCIF report

## Figures and Tables

**Table 1:** Hydrogen-bond geometry (Å, °)

*D*—H⋯*A*	*D*—H	H⋯*A*	*D*⋯*A*	*D*—H⋯*A*
N1—H1⋯O1	0.95 (3)	1.91 (3)	2.794 (4)	154 (3)
N2—H2⋯O10	0.87 (4)	2.04 (4)	2.821 (4)	149 (3)
O3—H31⋯O11^i^	0.91	1.89	2.700 (3)	147
O3—H32⋯O7	0.92	2.17	2.907 (3)	137
O4—H41⋯O9^i^	0.99	2.05	2.951 (3)	151
O4—H42⋯O1	0.99	1.91	2.726 (3)	138
O5—H52⋯O8^i^	0.85	1.96	2.741 (4)	153
O6—H61⋯O1^ii^	0.97	1.93	2.754 (4)	142
O7—H71⋯O10	0.98	1.86	2.751 (4)	148
O7—H72⋯O3^iii^	0.99	1.91	2.894 (4)	177
O8—H81⋯O11^iv^	0.88	1.89	2.760 (4)	170
O8—H82⋯O11	0.88	1.94	2.816 (4)	172
O9—H91⋯O5^ii^	0.89	1.98	2.850 (4)	165
O5—H51⋯Cl20^iii^	0.85	2.63	3.339 (3)	142

Symmetry codes: (i) 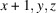; (ii) 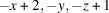; (iii) 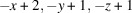; (iv) 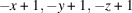.

**Table 2:** Experimental details

Crystal data	
Chemical formula	[Na_4_(C_14_H_10_Cl_2_NO_2_)_2_(H_2_O)_14_](C_14_H_10_Cl_2_NO_2_)_2_
*M*_r_	1524.70
Crystal system, space group	Triclinic, *P* 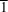
Temperature (K)	295
*a*, *b*, *c* (Å)	9.4370 (4), 9.5675 (5), 19.1526 (10)
α, β, γ (°)	90.331 (4), 99.828 (4), 90.436 (4)
*V* (Å^3^)	1703.79 (15)
*Z*	1
Radiation type	Ag *K*α, λ = 0.56083 Å
μ (mm^−1^)	0.23
Crystal size (mm)	0.18 × 0.18 × 0.06

Data collection	
Diffractometer	Stoe Stadivari
Absorption correction	Multi-scan (*X-AREA*; Stoe & Cie, 2019 ▸)
*T*_min_, *T*_max_	0.328, 1.000
No. of measured, independent and observed [*I* > 2σ(*I*)] reflections	51408, 6930, 3920
*R*_int_	0.101
(sin θ/λ)_max_ (Å^−1^)	0.625

Refinement	
*R*[*F* ^2^ > 2σ(*F* ^2^)], *wR*(*F* ^2^), *S*	0.041, 0.103, 0.84
No. of reflections	6930
No. of parameters	438
H-atom treatment	H atoms treated by a mixture of independent and constrained refinement
Δρ_max_, Δρ_min_ (e Å^−3^)	0.25, −0.23

Computer programs: *X-AREA* (Stoe & Cie, 2019 ▸), *SHELXT2018/2* (Sheldrick, 2015*a* ▸), *SHELXL2018/3* (Sheldrick, 2015*b* ▸), *XP* in *SHELXTL-Plus* (Sheldrick, 2008 ▸), *Mercury* (Macrae *et al.*, 2020 ▸) and *publCIF* (Westrip, 2010 ▸).

## supplementary crystallographic information

### Crystal data

**Table:**

[Na_4_(C_14_H_10_Cl_2_NO_2_)_2_(H_2_O)_14_](C_14_H_10_Cl_2_NO_2_)_2_	*Z* = 1
*M**_r_* = 1524.70	*F*(000) = 788
Triclinic, *P*1	*D*_x_ = 1.486 Mg m^−^^3^
*a* = 9.4370 (4) Å	Ag *K*α radiation, λ = 0.56083 Å
*b* = 9.5675 (5) Å	Cell parameters from 29092 reflections
*c* = 19.1526 (10) Å	θ = 2.4–22.6°
α = 90.331 (4)°	µ = 0.23 mm^−^^1^
β = 99.828 (4)°	*T* = 295 K
γ = 90.436 (4)°	Prism, yellow
*V* = 1703.79 (15) Å^3^	0.18 × 0.18 × 0.06 mm

### Data collection

**Table:**

Stoe Stadivari diffractometer	6930 independent reflections
Radiation source: Sealed X-ray tube, Axo Astix-f Microfocus source	3920 reflections with *I* > 2σ(*I*)
Graded multilayer mirror monochromator	*R*_int_ = 0.101
Detector resolution: 5.81 pixels mm^-1^	θ_max_ = 20.5°, θ_min_ = 2.4°
ω scans	*h* = −11→11
Absorption correction: multi-scan (X-AREA; Stoe & Cie, 2019)	*k* = −11→11
*T*_min_ = 0.328, *T*_max_ = 1.000	*l* = −23→23
51408 measured reflections	

### Refinement

**Table:**

Refinement on *F*^2^	Primary atom site location: dual
Least-squares matrix: full	Secondary atom site location: difference Fourier map
*R*[*F*^2^ > 2σ(*F*^2^)] = 0.041	Hydrogen site location: mixed
*wR*(*F*^2^) = 0.103	H atoms treated by a mixture of independent and constrained refinement
*S* = 0.84	*w* = 1/[σ^2^(*F*_o_^2^) + (0.048*P*)^2^] where *P* = (*F*_o_^2^ + 2*F*_c_^2^)/3
6930 reflections	(Δ/σ)_max_ = 0.001
438 parameters	Δρ_max_ = 0.25 e Å^−^^3^
0 restraints	Δρ_min_ = −0.23 e Å^−^^3^

### Special details

**Table:**

Refinement. Refined as a two-component twin

### Fractional atomic coordinates and isotropic or equivalent isotropic displacement parameters (Å^2^)

**Table:**

	*x*	*y*	*z*	*U*_iso_*/*U*_eq_	
N1	1.2170 (3)	0.0047 (3)	0.80000 (15)	0.0406 (7)	
H1	1.198 (3)	−0.029 (4)	0.7524 (18)	0.049*	
C1	1.3072 (3)	−0.0732 (3)	0.84998 (18)	0.0385 (8)	
C2	1.3117 (3)	−0.0611 (3)	0.92322 (18)	0.0397 (8)	
Cl2	1.19687 (9)	0.05130 (10)	0.95770 (5)	0.0492 (2)	
C3	1.4023 (3)	−0.1411 (4)	0.97186 (19)	0.0469 (9)	
H3	1.406005	−0.126940	1.020221	0.056*	
C4	1.4868 (4)	−0.2418 (4)	0.9479 (2)	0.0528 (10)	
H4	1.548903	−0.294917	0.979987	0.063*	
C5	1.4785 (4)	−0.2631 (4)	0.8764 (2)	0.0504 (9)	
H5	1.532216	−0.333466	0.860094	0.060*	
C6	1.3918 (3)	−0.1814 (4)	0.82915 (18)	0.0444 (8)	
Cl6	1.38649 (11)	−0.21441 (11)	0.73852 (5)	0.0592 (3)	
C7	1.2078 (3)	0.1532 (3)	0.80037 (17)	0.0369 (8)	
C8	1.3044 (3)	0.2359 (4)	0.84662 (18)	0.0417 (8)	
H8	1.378483	0.193739	0.877362	0.050*	
C9	1.2913 (4)	0.3793 (4)	0.84734 (19)	0.0467 (9)	
H9	1.354660	0.433064	0.879402	0.056*	
C10	1.1843 (4)	0.4432 (4)	0.8005 (2)	0.0507 (9)	
H10	1.174680	0.539862	0.800613	0.061*	
C11	1.0915 (4)	0.3608 (4)	0.75336 (19)	0.0461 (9)	
H11	1.020968	0.404066	0.721033	0.055*	
C12	1.0996 (3)	0.2164 (4)	0.75243 (17)	0.0385 (8)	
C13	0.9862 (3)	0.1324 (4)	0.70255 (17)	0.0444 (8)	
H13A	0.949019	0.059927	0.729863	0.053*	
H13B	0.907138	0.194079	0.685114	0.053*	
C14	1.0357 (4)	0.0637 (4)	0.63902 (19)	0.0437 (8)	
O1	1.1477 (3)	−0.0085 (3)	0.65231 (13)	0.0615 (7)	
O2	0.9652 (2)	0.0778 (3)	0.57822 (12)	0.0497 (6)	
N2	0.7304 (3)	0.4820 (3)	0.80106 (16)	0.0480 (8)	
H2	0.725 (4)	0.506 (4)	0.757 (2)	0.058*	
C15	0.8157 (3)	0.5640 (4)	0.85260 (18)	0.0398 (8)	
C16	0.8131 (3)	0.5537 (4)	0.92522 (18)	0.0406 (8)	
Cl16	0.69399 (9)	0.43983 (10)	0.95609 (5)	0.0521 (2)	
C17	0.8980 (4)	0.6382 (4)	0.9748 (2)	0.0497 (9)	
H17	0.895158	0.626748	1.022720	0.060*	
C18	0.9863 (4)	0.7386 (4)	0.9539 (2)	0.0517 (10)	
H18	1.045862	0.792692	0.987409	0.062*	
C19	0.9856 (4)	0.7586 (4)	0.8823 (2)	0.0500 (9)	
H19	1.040696	0.829867	0.867355	0.060*	
C20	0.9031 (3)	0.6725 (4)	0.83345 (18)	0.0413 (8)	
Cl20	0.90705 (11)	0.70100 (11)	0.74380 (5)	0.0598 (3)	
C21	0.7186 (3)	0.3338 (4)	0.80349 (17)	0.0398 (8)	
C22	0.8201 (4)	0.2535 (4)	0.84606 (18)	0.0445 (9)	
H22	0.900569	0.296260	0.872715	0.053*	
C23	0.8025 (4)	0.1105 (4)	0.8491 (2)	0.0507 (9)	
H23	0.869922	0.057350	0.878364	0.061*	
C24	0.6841 (4)	0.0462 (4)	0.8084 (2)	0.0579 (10)	
H24	0.671920	−0.050170	0.810366	0.069*	
C25	0.5850 (4)	0.1249 (4)	0.7652 (2)	0.0495 (9)	
H25	0.506577	0.080695	0.737643	0.059*	
C26	0.5992 (3)	0.2699 (4)	0.76160 (17)	0.0423 (8)	
C27	0.4867 (3)	0.3546 (4)	0.71529 (18)	0.0510 (9)	
H27A	0.456973	0.429112	0.744042	0.061*	
H27B	0.403464	0.294866	0.700275	0.061*	
C28	0.5293 (4)	0.4200 (4)	0.6495 (2)	0.0546 (10)	
O10	0.6558 (3)	0.4628 (4)	0.65228 (14)	0.0942 (12)	
O11	0.4341 (3)	0.4346 (3)	0.59671 (13)	0.0631 (7)	
Na1	1.11754 (13)	0.14829 (16)	0.48702 (8)	0.0547 (4)	
Na2	0.75703 (14)	0.20206 (16)	0.52183 (8)	0.0586 (4)	
O3	1.1555 (3)	0.3696 (3)	0.54845 (13)	0.0649 (7)	
H31	1.240945	0.371506	0.579090	0.097*	
H32	1.091820	0.384878	0.578875	0.097*	
O4	1.3116 (2)	0.0135 (3)	0.54862 (13)	0.0589 (7)	
H41	1.404768	0.064234	0.556123	0.088*	
H42	1.292129	−0.023543	0.594155	0.088*	
O5	1.2920 (3)	0.2037 (3)	0.40943 (14)	0.0739 (8)	
H51	1.276724	0.206890	0.364317	0.111*	
H52	1.380780	0.224266	0.421379	0.111*	
O6	0.8992 (3)	0.2519 (3)	0.42599 (14)	0.0678 (8)	
H61	0.850781	0.193978	0.387203	0.102*	
H62	0.914050	0.345665	0.409367	0.102*	
O7	0.8675 (3)	0.4414 (3)	0.57032 (15)	0.0765 (9)	
H71	0.814665	0.477381	0.606679	0.115*	
H72	0.859682	0.508255	0.530947	0.115*	
O8	0.5406 (2)	0.3249 (3)	0.47928 (14)	0.0605 (7)	
H81	0.557848	0.403845	0.458558	0.091*	
H82	0.500368	0.353551	0.514975	0.091*	
O9	0.6181 (2)	0.0786 (3)	0.59983 (14)	0.0684 (8)	
H91	0.644528	−0.010265	0.605026	0.103*	
H92	0.637711	0.111890	0.643827	0.103*	

### Atomic displacement parameters (Å^2^)

**Table:**

	*U*^11^	*U*^22^	*U*^33^	*U*^12^	*U*^13^	*U*^23^
N1	0.0489 (16)	0.0334 (17)	0.0375 (16)	0.0050 (13)	0.0014 (14)	−0.0027 (13)
C1	0.0402 (18)	0.0306 (19)	0.044 (2)	−0.0023 (15)	0.0047 (16)	0.0029 (16)
C2	0.0412 (17)	0.0313 (19)	0.046 (2)	−0.0029 (15)	0.0057 (16)	−0.0027 (16)
Cl2	0.0563 (5)	0.0463 (5)	0.0466 (5)	0.0033 (4)	0.0130 (4)	−0.0025 (4)
C3	0.052 (2)	0.044 (2)	0.043 (2)	−0.0045 (18)	0.0009 (17)	0.0047 (17)
C4	0.051 (2)	0.043 (2)	0.061 (3)	0.0057 (18)	−0.0010 (19)	0.0123 (19)
C5	0.046 (2)	0.036 (2)	0.070 (3)	0.0038 (17)	0.010 (2)	0.0042 (19)
C6	0.0450 (18)	0.040 (2)	0.049 (2)	0.0005 (17)	0.0099 (17)	0.0005 (17)
Cl6	0.0717 (6)	0.0536 (6)	0.0534 (6)	0.0122 (5)	0.0130 (5)	−0.0079 (5)
C7	0.0409 (17)	0.035 (2)	0.0363 (18)	0.0006 (15)	0.0114 (15)	−0.0011 (15)
C8	0.0411 (18)	0.043 (2)	0.041 (2)	0.0004 (16)	0.0062 (16)	0.0046 (16)
C9	0.050 (2)	0.041 (2)	0.051 (2)	−0.0078 (17)	0.0117 (18)	−0.0069 (18)
C10	0.057 (2)	0.034 (2)	0.063 (3)	0.0026 (18)	0.016 (2)	−0.0019 (19)
C11	0.0482 (19)	0.044 (2)	0.047 (2)	0.0093 (17)	0.0096 (17)	0.0008 (17)
C12	0.0397 (17)	0.039 (2)	0.0368 (19)	0.0054 (16)	0.0082 (15)	−0.0020 (16)
C13	0.0408 (18)	0.051 (2)	0.040 (2)	0.0062 (16)	0.0037 (15)	−0.0036 (17)
C14	0.0466 (19)	0.046 (2)	0.040 (2)	0.0004 (17)	0.0106 (17)	−0.0045 (17)
O1	0.0640 (15)	0.078 (2)	0.0394 (14)	0.0325 (15)	0.0005 (12)	−0.0085 (13)
O2	0.0500 (13)	0.0588 (16)	0.0368 (14)	0.0058 (12)	−0.0031 (11)	−0.0031 (12)
N2	0.0561 (17)	0.047 (2)	0.0383 (17)	−0.0033 (15)	0.0005 (15)	0.0054 (15)
C15	0.0392 (17)	0.0317 (19)	0.048 (2)	0.0065 (15)	0.0044 (16)	0.0028 (16)
C16	0.0459 (18)	0.0329 (19)	0.043 (2)	0.0042 (16)	0.0079 (16)	0.0039 (16)
Cl16	0.0584 (5)	0.0468 (6)	0.0538 (6)	−0.0013 (4)	0.0171 (4)	0.0056 (4)
C17	0.055 (2)	0.045 (2)	0.048 (2)	0.0077 (19)	0.0072 (18)	−0.0026 (18)
C18	0.049 (2)	0.044 (2)	0.059 (3)	0.0000 (18)	0.0006 (19)	−0.0097 (19)
C19	0.0457 (19)	0.042 (2)	0.063 (3)	−0.0033 (17)	0.0125 (19)	0.0000 (19)
C20	0.0430 (18)	0.038 (2)	0.044 (2)	0.0077 (16)	0.0095 (16)	0.0072 (16)
Cl20	0.0672 (6)	0.0614 (7)	0.0522 (6)	−0.0018 (5)	0.0133 (5)	0.0150 (5)
C21	0.0446 (18)	0.040 (2)	0.0355 (19)	−0.0029 (16)	0.0075 (16)	−0.0005 (16)
C22	0.0447 (19)	0.049 (2)	0.039 (2)	0.0019 (17)	0.0050 (16)	−0.0021 (17)
C23	0.058 (2)	0.041 (2)	0.055 (2)	0.0121 (19)	0.015 (2)	0.0039 (19)
C24	0.070 (3)	0.037 (2)	0.071 (3)	−0.002 (2)	0.026 (2)	−0.004 (2)
C25	0.048 (2)	0.051 (2)	0.052 (2)	−0.0082 (18)	0.0145 (18)	−0.0071 (19)
C26	0.0397 (17)	0.052 (2)	0.0363 (19)	−0.0019 (16)	0.0093 (15)	0.0018 (17)
C27	0.0446 (19)	0.069 (3)	0.039 (2)	−0.0103 (19)	0.0063 (17)	0.0065 (19)
C28	0.049 (2)	0.071 (3)	0.043 (2)	−0.007 (2)	0.0067 (19)	0.005 (2)
O10	0.0542 (16)	0.180 (4)	0.0468 (17)	−0.030 (2)	0.0038 (14)	0.025 (2)
O11	0.0518 (14)	0.085 (2)	0.0482 (16)	−0.0089 (14)	−0.0026 (13)	0.0148 (15)
Na1	0.0478 (7)	0.0577 (9)	0.0579 (9)	0.0024 (7)	0.0069 (7)	−0.0037 (7)
Na2	0.0516 (8)	0.0607 (10)	0.0620 (9)	0.0100 (7)	0.0056 (7)	−0.0055 (8)
O3	0.0508 (14)	0.080 (2)	0.0633 (18)	−0.0057 (14)	0.0076 (13)	−0.0045 (15)
O4	0.0511 (13)	0.0724 (19)	0.0525 (16)	0.0029 (13)	0.0068 (12)	−0.0015 (14)
O5	0.0659 (17)	0.096 (2)	0.0593 (17)	−0.0008 (17)	0.0080 (14)	0.0157 (18)
O6	0.0749 (17)	0.0581 (18)	0.0662 (18)	0.0027 (15)	0.0004 (15)	−0.0042 (14)
O7	0.0770 (18)	0.090 (2)	0.0682 (19)	0.0322 (17)	0.0273 (16)	0.0100 (17)
O8	0.0638 (16)	0.0562 (17)	0.0635 (18)	0.0096 (14)	0.0160 (14)	0.0051 (14)
O9	0.0574 (15)	0.086 (2)	0.0624 (17)	0.0021 (15)	0.0130 (13)	−0.0121 (16)

### Geometric parameters (Å, º)

**Table:**

N1—C1	1.391 (4)	C19—H19	0.9300
N1—C7	1.425 (4)	C20—Cl20	1.747 (3)
N1—H1	0.95 (3)	C21—C22	1.388 (5)
C1—C2	1.400 (4)	C21—C26	1.400 (5)
C1—C6	1.409 (4)	C22—C23	1.379 (5)
C2—C3	1.390 (5)	C22—H22	0.9300
C2—Cl2	1.738 (3)	C23—C24	1.386 (5)
C3—C4	1.381 (5)	C23—H23	0.9300
C3—H3	0.9300	C24—C25	1.371 (5)
C4—C5	1.371 (5)	C24—H24	0.9300
C4—H4	0.9300	C25—C26	1.396 (5)
C5—C6	1.366 (5)	C25—H25	0.9300
C5—H5	0.9300	C26—C27	1.507 (5)
C6—Cl6	1.755 (4)	C27—C28	1.523 (5)
C7—C8	1.395 (4)	C27—H27A	0.9700
C7—C12	1.395 (4)	C27—H27B	0.9700
C8—C9	1.379 (5)	C28—O11	1.242 (4)
C8—H8	0.9300	C28—O10	1.251 (4)
C9—C10	1.380 (5)	Na1—O4	2.393 (3)
C9—H9	0.9300	Na1—O3	2.411 (3)
C10—C11	1.384 (5)	Na1—O6	2.411 (3)
C10—H10	0.9300	Na1—O5	2.455 (3)
C11—C12	1.384 (5)	Na1—Na2^i^	3.576 (2)
C11—H11	0.9300	Na1—Na2	3.6167 (18)
C12—C13	1.527 (5)	Na1—Na1^i^	3.677 (3)
C13—C14	1.523 (4)	Na2—O8	2.387 (3)
C13—H13A	0.9700	Na2—O9	2.452 (3)
C13—H13B	0.9700	Na2—O4^i^	2.479 (3)
C14—O2	1.247 (4)	Na2—O6	2.498 (3)
C14—O1	1.257 (4)	Na2—O7	2.608 (4)
O2—Na2	2.401 (3)	O3—H31	0.9125
O2—Na1	2.535 (3)	O3—H32	0.9177
O2—Na1^i^	2.542 (3)	O4—H41	0.9889
N2—C15	1.396 (4)	O4—H42	0.9889
N2—C21	1.423 (4)	O5—H51	0.8522
N2—H2	0.87 (4)	O5—H52	0.8517
C15—C16	1.399 (5)	O6—H61	0.9711
C15—C20	1.410 (5)	O6—H62	0.9711
C16—C17	1.385 (5)	O7—H71	0.9849
C16—Cl16	1.736 (3)	O7—H72	0.9851
C17—C18	1.373 (5)	O8—H81	0.8821
C17—H17	0.9300	O8—H82	0.8801
C18—C19	1.383 (5)	O9—H91	0.8890
C18—H18	0.9300	O9—H92	0.8881
C19—C20	1.376 (5)		
			
C1—N1—C7	124.8 (3)	C28—C27—H27B	108.1
C1—N1—H1	118 (2)	H27A—C27—H27B	107.3
C7—N1—H1	110 (2)	O11—C28—O10	123.3 (4)
N1—C1—C2	123.9 (3)	O11—C28—C27	117.9 (3)
N1—C1—C6	121.1 (3)	O10—C28—C27	118.8 (3)
C2—C1—C6	114.7 (3)	O4—Na1—O3	101.57 (10)
C3—C2—C1	122.6 (3)	O4—Na1—O6	171.04 (11)
C3—C2—Cl2	116.4 (3)	O3—Na1—O6	85.00 (10)
C1—C2—Cl2	120.9 (3)	O4—Na1—O5	83.10 (9)
C4—C3—C2	119.5 (3)	O3—Na1—O5	92.81 (10)
C4—C3—H3	120.2	O6—Na1—O5	102.83 (10)
C2—C3—H3	120.2	O4—Na1—O2	89.51 (9)
C5—C4—C3	119.6 (3)	O3—Na1—O2	87.16 (9)
C5—C4—H4	120.2	O6—Na1—O2	84.69 (9)
C3—C4—H4	120.2	O5—Na1—O2	172.45 (10)
C6—C5—C4	120.3 (3)	O4—Na1—O2^i^	85.65 (9)
C6—C5—H5	119.8	O3—Na1—O2^i^	170.80 (10)
C4—C5—H5	119.8	O6—Na1—O2^i^	87.25 (9)
C5—C6—C1	123.0 (3)	O5—Na1—O2^i^	93.72 (10)
C5—C6—Cl6	117.9 (3)	O2—Na1—O2^i^	87.21 (9)
C1—C6—Cl6	119.1 (3)	O4—Na1—Na2^i^	43.71 (7)
C8—C7—C12	119.7 (3)	O3—Na1—Na2^i^	145.27 (8)
C8—C7—N1	121.8 (3)	O6—Na1—Na2^i^	129.35 (9)
C12—C7—N1	118.5 (3)	O5—Na1—Na2^i^	84.90 (8)
C9—C8—C7	120.8 (3)	O2—Na1—Na2^i^	90.83 (7)
C9—C8—H8	119.6	O2^i^—Na1—Na2^i^	42.11 (6)
C7—C8—H8	119.6	O4—Na1—Na2	130.95 (8)
C8—C9—C10	120.0 (3)	O3—Na1—Na2	81.17 (7)
C8—C9—H9	120.0	O6—Na1—Na2	43.48 (7)
C10—C9—H9	120.0	O5—Na1—Na2	145.96 (9)
C9—C10—C11	118.8 (3)	O2—Na1—Na2	41.45 (6)
C9—C10—H10	120.6	O2^i^—Na1—Na2	89.80 (7)
C11—C10—H10	120.6	Na2^i^—Na1—Na2	118.52 (4)
C10—C11—C12	122.6 (3)	O4—Na1—Na1^i^	86.65 (8)
C10—C11—H11	118.7	O3—Na1—Na1^i^	130.42 (9)
C12—C11—H11	118.7	O6—Na1—Na1^i^	84.43 (8)
C11—C12—C7	118.0 (3)	O5—Na1—Na1^i^	136.77 (10)
C11—C12—C13	119.4 (3)	O2—Na1—Na1^i^	43.68 (6)
C7—C12—C13	122.6 (3)	O2^i^—Na1—Na1^i^	43.54 (6)
C14—C13—C12	116.4 (3)	Na2^i^—Na1—Na1^i^	59.80 (5)
C14—C13—H13A	108.2	Na2—Na1—Na1^i^	58.72 (4)
C12—C13—H13A	108.2	O8—Na2—O2	173.08 (11)
C14—C13—H13B	108.2	O8—Na2—O9	86.20 (10)
C12—C13—H13B	108.2	O2—Na2—O9	87.99 (10)
H13A—C13—H13B	107.3	O8—Na2—O4^i^	95.71 (10)
O2—C14—O1	123.4 (3)	O2—Na2—O4^i^	86.90 (9)
O2—C14—C13	120.5 (3)	O9—Na2—O4^i^	79.41 (10)
O1—C14—C13	116.0 (3)	O8—Na2—O6	100.91 (10)
C14—O2—Na2	138.1 (2)	O2—Na2—O6	85.74 (9)
C14—O2—Na1	113.5 (2)	O9—Na2—O6	161.78 (11)
Na2—O2—Na1	94.18 (10)	O4^i^—Na2—O6	83.18 (10)
C14—O2—Na1^i^	115.4 (2)	O8—Na2—O7	87.19 (10)
Na2—O2—Na1^i^	92.64 (9)	O2—Na2—O7	91.90 (9)
Na1—O2—Na1^i^	92.79 (9)	O9—Na2—O7	115.21 (10)
C15—N2—C21	124.6 (3)	O4^i^—Na2—O7	165.30 (11)
C15—N2—H2	118 (3)	O6—Na2—O7	82.12 (10)
C21—N2—H2	108 (3)	O8—Na2—Na1^i^	136.50 (9)
N2—C15—C16	123.7 (3)	O2—Na2—Na1^i^	45.24 (7)
N2—C15—C20	121.0 (3)	O9—Na2—Na1^i^	78.02 (8)
C16—C15—C20	115.1 (3)	O4^i^—Na2—Na1^i^	41.84 (6)
C17—C16—C15	122.1 (3)	O6—Na2—Na1^i^	85.46 (8)
C17—C16—Cl16	117.3 (3)	O7—Na2—Na1^i^	136.20 (7)
C15—C16—Cl16	120.5 (3)	O8—Na2—Na1	142.04 (9)
C18—C17—C16	120.6 (3)	O2—Na2—Na1	44.36 (6)
C18—C17—H17	119.7	O9—Na2—Na1	131.19 (8)
C16—C17—H17	119.7	O4^i^—Na2—Na1	86.78 (7)
C17—C18—C19	119.3 (4)	O6—Na2—Na1	41.63 (7)
C17—C18—H18	120.4	O7—Na2—Na1	82.18 (7)
C19—C18—H18	120.4	Na1^i^—Na2—Na1	61.48 (4)
C20—C19—C18	119.7 (3)	Na1—O3—H31	111.3
C20—C19—H19	120.2	Na1—O3—H32	113.0
C18—C19—H19	120.2	H31—O3—H32	101.1
C19—C20—C15	123.0 (3)	Na1—O4—Na2^i^	94.44 (9)
C19—C20—Cl20	118.0 (3)	Na1—O4—H41	113.2
C15—C20—Cl20	119.1 (3)	Na2^i^—O4—H41	128.5
C22—C21—C26	120.3 (3)	Na1—O4—H42	113.0
C22—C21—N2	121.9 (3)	Na2^i^—O4—H42	96.3
C26—C21—N2	117.8 (3)	H41—O4—H42	110.0
C23—C22—C21	120.4 (3)	Na1—O5—H51	127.5
C23—C22—H22	119.8	Na1—O5—H52	128.0
C21—C22—H22	119.8	H51—O5—H52	104.5
C22—C23—C24	119.9 (4)	Na1—O6—Na2	94.89 (10)
C22—C23—H23	120.0	Na1—O6—H61	112.7
C24—C23—H23	120.0	Na2—O6—H61	102.3
C25—C24—C23	119.9 (4)	Na1—O6—H62	112.9
C25—C24—H24	120.1	Na2—O6—H62	122.9
C23—C24—H24	120.1	H61—O6—H62	110.2
C24—C25—C26	121.5 (4)	Na2—O7—H71	109.7
C24—C25—H25	119.2	Na2—O7—H72	109.0
C26—C25—H25	119.2	H71—O7—H72	109.4
C25—C26—C21	118.1 (3)	Na2—O8—H81	111.7
C25—C26—C27	120.5 (3)	Na2—O8—H82	110.4
C21—C26—C27	121.4 (3)	H81—O8—H82	102.7
C26—C27—C28	117.0 (3)	Na2—O9—H91	111.1
C26—C27—H27A	108.1	Na2—O9—H92	111.2
C28—C27—H27A	108.1	H91—O9—H92	102.8
C26—C27—H27B	108.1		
			
C7—N1—C1—C2	53.3 (4)	C13—C14—O2—Na1	131.2 (3)
C7—N1—C1—C6	−133.2 (3)	O1—C14—O2—Na1^i^	55.1 (4)
N1—C1—C2—C3	179.5 (3)	C13—C14—O2—Na1^i^	−123.3 (3)
C6—C1—C2—C3	5.6 (4)	C21—N2—C15—C16	−50.2 (5)
N1—C1—C2—Cl2	2.3 (4)	C21—N2—C15—C20	135.1 (3)
C6—C1—C2—Cl2	−171.7 (2)	N2—C15—C16—C17	−179.1 (3)
C1—C2—C3—C4	−3.3 (5)	C20—C15—C16—C17	−4.2 (5)
Cl2—C2—C3—C4	174.0 (3)	N2—C15—C16—Cl16	−2.9 (4)
C2—C3—C4—C5	−1.0 (5)	C20—C15—C16—Cl16	172.0 (2)
C3—C4—C5—C6	2.7 (5)	C15—C16—C17—C18	1.6 (5)
C4—C5—C6—C1	−0.1 (5)	Cl16—C16—C17—C18	−174.7 (3)
C4—C5—C6—Cl6	180.0 (3)	C16—C17—C18—C19	2.5 (5)
N1—C1—C6—C5	−178.0 (3)	C17—C18—C19—C20	−3.7 (5)
C2—C1—C6—C5	−3.9 (5)	C18—C19—C20—C15	1.0 (5)
N1—C1—C6—Cl6	1.9 (4)	C18—C19—C20—Cl20	−179.5 (3)
C2—C1—C6—Cl6	176.0 (2)	N2—C15—C20—C19	178.0 (3)
C1—N1—C7—C8	8.8 (5)	C16—C15—C20—C19	2.9 (5)
C1—N1—C7—C12	−171.8 (3)	N2—C15—C20—Cl20	−1.5 (4)
C12—C7—C8—C9	2.3 (5)	C16—C15—C20—Cl20	−176.6 (2)
N1—C7—C8—C9	−178.3 (3)	C15—N2—C21—C22	−18.9 (5)
C7—C8—C9—C10	−1.9 (5)	C15—N2—C21—C26	160.6 (3)
C8—C9—C10—C11	0.0 (5)	C26—C21—C22—C23	−1.8 (5)
C9—C10—C11—C12	1.6 (5)	N2—C21—C22—C23	177.6 (3)
C10—C11—C12—C7	−1.2 (5)	C21—C22—C23—C24	1.2 (5)
C10—C11—C12—C13	175.4 (3)	C22—C23—C24—C25	0.1 (5)
C8—C7—C12—C11	−0.7 (4)	C23—C24—C25—C26	−0.8 (5)
N1—C7—C12—C11	179.9 (3)	C24—C25—C26—C21	0.2 (5)
C8—C7—C12—C13	−177.2 (3)	C24—C25—C26—C27	−178.3 (3)
N1—C7—C12—C13	3.4 (4)	C22—C21—C26—C25	1.1 (5)
C11—C12—C13—C14	108.4 (4)	N2—C21—C26—C25	−178.4 (3)
C7—C12—C13—C14	−75.2 (4)	C22—C21—C26—C27	179.5 (3)
C12—C13—C14—O2	−130.6 (3)	N2—C21—C26—C27	0.1 (4)
C12—C13—C14—O1	50.8 (4)	C25—C26—C27—C28	−109.9 (4)
O1—C14—O2—Na2	−177.5 (2)	C21—C26—C27—C28	71.7 (4)
C13—C14—O2—Na2	4.0 (5)	C26—C27—C28—O11	148.7 (4)
O1—C14—O2—Na1	−50.3 (4)	C26—C27—C28—O10	−35.0 (6)

Symmetry code: (i) −*x*+2, −*y*, −*z*+1.

### Hydrogen-bond geometry (Å, º)

**Table:**

*D*—H···*A*	*D*—H	H···*A*	*D*···*A*	*D*—H···*A*
N1—H1···O1	0.95 (3)	1.91 (3)	2.794 (4)	154 (3)
N2—H2···O10	0.87 (4)	2.04 (4)	2.821 (4)	149 (3)
O3—H31···O11^ii^	0.91	1.89	2.700 (3)	147
O3—H32···O7	0.92	2.17	2.907 (3)	137
O4—H41···O9^ii^	0.99	2.05	2.951 (3)	151
O4—H42···O1	0.99	1.91	2.726 (3)	138
O5—H52···O8^ii^	0.85	1.96	2.741 (4)	153
O6—H61···O1^i^	0.97	1.93	2.754 (4)	142
O7—H71···O10	0.98	1.86	2.751 (4)	148
O7—H72···O3^iii^	0.99	1.91	2.894 (4)	177
O8—H81···O11^iv^	0.88	1.89	2.760 (4)	170
O8—H82···O11	0.88	1.94	2.816 (4)	172
O9—H91···O5^i^	0.89	1.98	2.850 (4)	165
O5—H51···Cl20^iii^	0.85	2.63	3.339 (3)	142

Symmetry codes: (i) −*x*+2, −*y*, −*z*+1; (ii) *x*+1, *y*, *z*; (iii) −*x*+2, −*y*+1, −*z*+1; (iv) −*x*+1, −*y*+1, −*z*+1.
